# Applications of Computational Intelligence in Time Series

**DOI:** 10.1155/2017/9361749

**Published:** 2017-05-22

**Authors:** Francisco Martínez-Álvarez, Alicia Troncoso, Jorge Reyes, María Martínez-Ballesteros, José C. Riquelme

**Affiliations:** ^1^Division of Computer Science, Pablo de Olavide University, 41013 Seville, Spain; ^2^NT2 Labs, Santiago, Chile; ^3^Department of Computer Science, University of Seville, Sevilla, Spain

The prediction of the future has fascinated the human being since its early existence. Actually, many of these efforts can be noticed in everyday events such as energy management, telecommunications, pollution, bioinformatics, and seismology and, obviously, in neuroscience. Accurate predictions are essential in economic activities as remarkable forecasting errors in certain areas may involve large loss of money.

In this context, the successful analysis of temporal data has been a challenging task for many researchers during the last decades and, indeed, it is difficult to figure out any scientific branch with no time-dependent variables.

Computational intelligence is known for including powerful techniques such as artificial neural networks, fuzzy systems, evolutionary computation, learning theory, or probabilistic methods. Thus, this special issue has been focused on the application of such techniques to time series. In particular, the goal was sharing recent advances in time series analysis and providing an interesting opportunity to present and discuss the latest practical advances in real-world applications.

Rigorous and extensive review processes have been carried out. Papers selected for this special issue present new findings and insights in the field of time series forecasting. A broad range of topics has been discussed, especially in the following areas: finances, tourism, feed grain demand, haze episodes, stock price, or data storage.

Genetic algorithms have been developed with binary coding to analyze high-speed trading research using price data of stocks on the microscopic level. This problem is certainly new and unexplored from computational intelligence techniques. Reported results show that the system is able to improve the accuracy for price movement forecasting, thus encouraging conducting research in this direction.

Tourism demand forecasting has also been addressed by means of seasonal trend autoregressive integrated moving averages with dendritic neural networks. Data from Japan are used to test the predictive performance of the proposed model. The model generates short-term predictions, after applying SARIMA models to exclude the long-term linear trend. This study mixes linear and nonlinear models and suggests that further analysis in the combination of such techniques is desirable.

Likewise, a new irregular sampling estimation method to extract the main trend of the time series has been proposed. To achieve this, first, the Kalman filter is used to remove dirty data. Second, the cubic spline interpolation and average method are used to reconstruct the main trend. The proposed approach has been applied to storage volume series of Internet data center. Results are quite promising.

A novel hybrid artificial intelligent system has been proposed to forecast stock price index trend. In particular, artificial neural networks combined with genetic algorithms have been applied to data from Thailand's SET50 index trend, from years 2009 to 2014. Multiple features and different time spans have been considered. Comparisons to other methods confirm the success of this novel methodology.

The long-term prediction of feed grain demand issue has been extensively discussed. A multivariate regression model along with a dynamic forecasting model has been introduced. Firstly, the correlation between the demand and its influence factors are studied and, secondly, changes in trend in factors affecting the demand are forecasted. Reported results corroborate the effectiveness of the methodology.

A novel long-term prediction model for Beijing haze episodes has been introduced. The authors have built a dynamic structural measurement model of daily haze increment and have reduced the model to a vector autoregressive model. Such model performs satisfactorily on next day's air quality index forecasting, reaching in many cases an accurate rate close to 90%. Therefore, sudden haze burst could be predicted with this method.

